# A deep learning framework to stratify Nottingham histologic grade 2 breast tumors based on dynamic contrast-enhanced MRI

**DOI:** 10.1007/s00330-025-12208-6

**Published:** 2025-12-17

**Authors:** Roham Hadidchi, Anchita Agrawal, Michael Z. Liu, Takouhie Maldijan, Yihui Zhu, Hien Quang Nguyen, Jinyu Lu, Della Makower, Susan Fineberg, Tim Q. Duong

**Affiliations:** 1 Department of Radiology, Montefiore Health System and Albert Einstein College of Medicine, Bronx, NY USA; 2 Department of Oncology, Montefiore Health System and Albert Einstein College of Medicine, Bronx, NY USA; 3 Department of Pathology, Montefiore Health System and Albert Einstein College of Medicine, Bronx, NY USA

**Keywords:** Breast neoplasms, Nottingham histologic grade, Risk stratification, Magnetic resonance imaging, Dynamic contrast-enhanced

## Abstract

**Objective:**

The Nottingham Histologic Grade (NHG) informs prognosis and treatment decisions in breast cancer, but NHG2 tumors are biologically heterogeneous, leading to both under- and over-treatment.

**Materials and methods:**

Clinical and imaging data from the Duke-Breast-Cancer-MRI (*n* = 877) and advanced-MRI-breast-lesions (*n* = 37) datasets were used to develop DeepRadGrade (DRG), a convolutional neural network model trained to differentiate NHG1 from NHG3 tumors on dynamic contrast-enhanced (DCE) MRI. The model then classified 456 NHG2 tumors into DRG2− (NHG1-like) and DRG2+ (NHG3-like) subgroups. Recurrence-free survival (RFS) was assessed with Kaplan–Meier and Cox models adjusting for age, lymph node invasion, tumor stage, and molecular subtype.

**Results:**

DRG achieved an AUC of 0.84 [95% CI: 0.83–0.86] in training, 0.82 [0.71–0.91] in testing, and 0.84 [0.69–0.96] in external validation. Among NHG2 tumors, 315 were classified as DRG2− and 131 as DRG2+. Patients with DRG2+ tumors had significantly worse RFS compared to DRG2− (adjusted hazard ratio = 2.39 [95% CI: 1.29–4.45], *p* = 0.0059), independent of standard prognostic factors. Incorporating DRG classification improved the Cox model’s *C*-index from 0.68 to 0.73 (*p* = 0.040).

**Conclusions:**

A deep learning model applied to routine DCE MRI effectively stratified NHG2 breast tumors into clinically meaningful subgroups with distinct recurrence risk. This approach offers a cost-effective tool for individualized risk stratification and could help tailor treatment to minimize over- and under-treatment in intermediate-grade breast cancer.

**Key Points:**

***Question*** *Difficulty in deciding between treatment options (neoadjuvant chemotherapy or primary surgery) in patients with intermediate risk breast cancer (Nottingham Grade 2).*

***Findings*** *Deep learning model reclassified grade 2 tumors into grade 1- and 3-like based on MRI. Patients with grade 3-like tumors had worse RFS: adjusted hazard ratio = 2.39 [95% CI: 1.29–4.45], p = 0.0059)*.

***Clinical relevance*** *Risk stratification could inform treatment choice to minimize over- and under-treatment in patients with intermediate-risk breast cancer*.

**Graphical Abstract:**

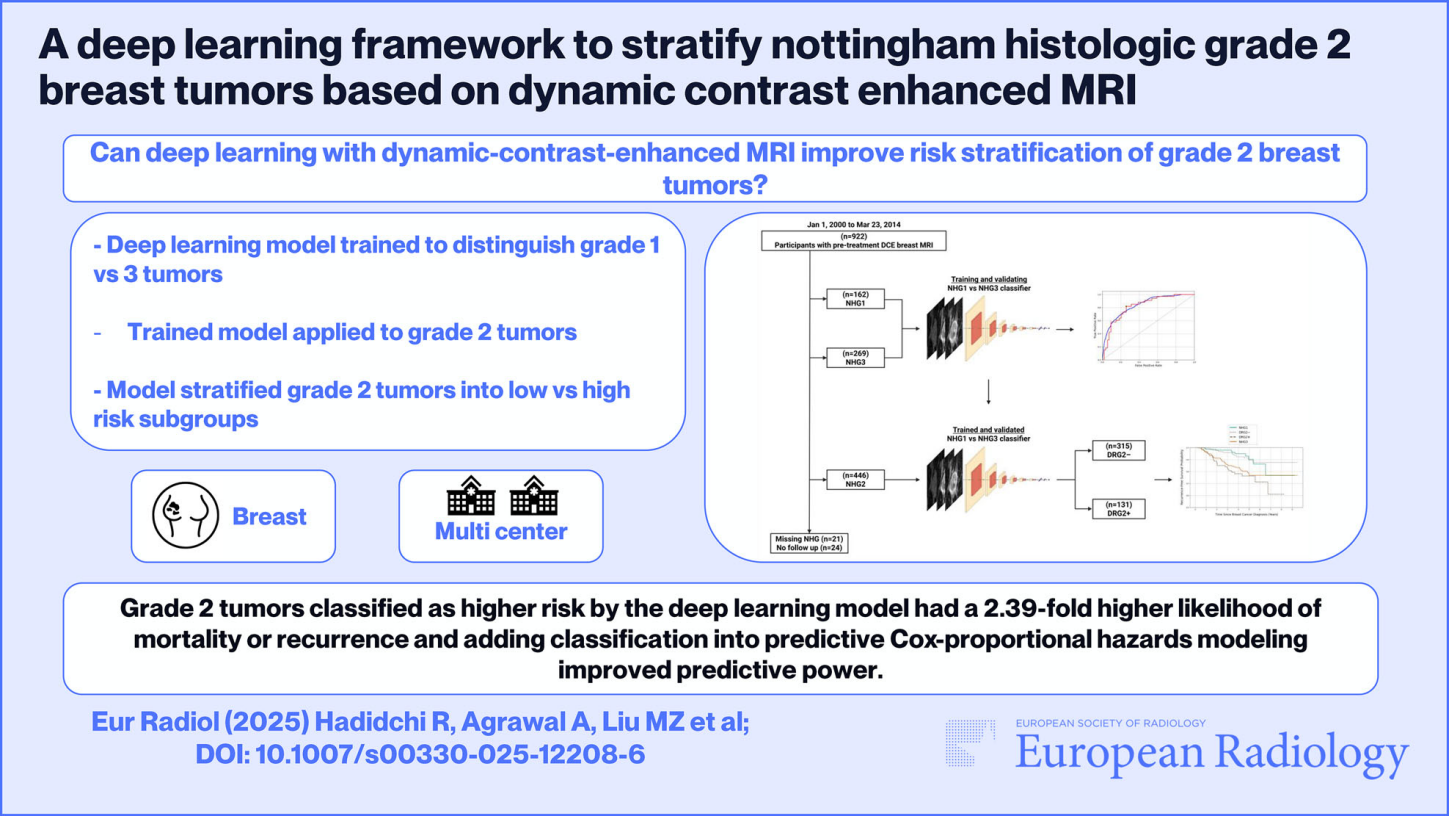

## Introduction

Among the various prognostic indicators in breast cancer, such as molecular subtype, staging, and patient age, the Nottingham Histologic Grade (NHG) stands as a central factor in shaping treatment decisions. NHG evaluates key histopathological features, including tubule formation, nuclear pleomorphism, and mitotic rate, assigning tumors a grade of NHG1 (well-differentiated), NHG2 (moderately differentiated), or NHG3 (poorly differentiated) [1]. Higher grades typically indicate more aggressive tumor behavior and are associated with worse prognosis [2].

NHG2, which accounts for half of all breast cancer cases [3–5], represents a heterogeneous group with diverse biological behaviors, various recurrence risk, and clinical outcomes. This variability contributes to uncertainty in treatment decisions, increasing the risk of both overtreatment and undertreatment [6]. Consequently, there is a growing need for improved risk stratification and more precise prognostic assessments to guide personalized treatment planning for patients with NHG2 breast cancer.

Several gene expression and genomic profiling techniques have been utilized to subdivide NHG2 tumors into high-risk and low-risk groups [7–10]. However, these molecular methods can be costly and time-consuming, limiting routine clinical application. Deep learning (DL) models, which excel in complex image pattern recognition, have emerged as a promising alternative for risk stratification [11–13]. Notably, one study demonstrated that a DL model using histopathological images could further subdivide NHG2 tumors into two distinct prognostic subgroups [14].

DL-based approaches have also been successfully applied to breast magnetic resonance imaging (MRI) for risk stratification across various outcomes, including the likelihood of breast malignancy, pathologic complete response, and Oncotype recurrence score [15–17]. However, their potential in refining risk assessment specifically for patients with NHG2 tumors remains largely unexplored. MRI is a non-invasive modality that provides three-dimensional visualization of breast tumors, offering high-resolution data on size, morphology, enhancement, and kinetic parameters that reflect underlying tumor biology [18]. Dynamic contrast-enhanced (DCE) MRI, which captures temporal changes in contrast uptake and washout, has proven to be highly valuable in characterizing tumor vascularity and perfusion, known indicators of tumor aggressiveness [19–21]. Leveraging routinely acquired DCE MRI data, DL-based risk stratification using DCE MRI enables a comprehensive, tumor-wide assessment at no additional cost. This approach has the potential to enhance prognostic accuracy and guide more personalized treatment decisions, ensuring that patients with higher-risk NHG2 tumors receive intensified therapy to reduce recurrence risk, while those with lower-risk tumors avoid overtreatment and its associated toxicities [22, 23].

Building on prior genomic profiling and histopathological studies, we developed and validated a DL framework using DCE MRI to refine risk stratification in breast cancer prognosis. First, we trained a DL model based on DCE MRI to classify tumors into NHG1 or NHG3. After external validation, we applied this model to DCE MRIs of NHG2 tumors, reclassifying them into NHG1-like or NHG3-like subgroups. We compared recurrence-free survival (RFS) between the two NHG2 subgroups. Additionally, we assessed whether this DL-based approach provides additional prognostic value independent of conventional clinical predictors, including age, lymph node invasion, tumor stage, and molecular subtype.

## Materials and methods

### Data sources

Data was obtained from the publicly available Duke-Breast-Cancer-MRI dataset [24, 25], which contains DCE breast MRI and other clinical data (such as age at diagnosis, staging, molecular subtype, and tubular formation, nuclear pleomorphism, and mitotic count) at diagnosis of 922 de-identified patients with biopsy-confirmed invasive breast cancer. NHG ground truth was calculated by summing the tubular formation, nuclear pleomorphism, and mitotic count of each tumor. Scores of 3–5 were classified as NHG1, 6 or 7 as NHG2, and 8 or 9 as NHG3. Patients with missing NHG scores (*n* = 21) were excluded. Patients (*n* = 24) who had no follow-up data were excluded from sub-stratification and RFS analysis.

Data was also obtained from the publicly available advanced-MRI-breast-lesions dataset [26], which contains DCE breast MRI and other clinical data at diagnosis of 200 de-identified patients with breast lesions. Those without breast cancer (only benign breast lesions), without MRI at diagnosis (only post-treatment MRI), or missing NHG scores (*n* = 123) were excluded. Stratification of NHG2 patients and RFS analysis were not performed on this dataset, as RFS follow-up data were not available.

### MRI acquisition

One pre-contrast and three or four post-contrast multi-slice T1-weighted fat-saturated gradient-echo phases were acquired axially using a Siemens or General Electric 1.5-T or 3-T scanner at a slice thickness of 1.00–2.5 mm and an in-plane resolution of 0.56–1.30 mm with contrast agent being administered intravenously via peripheral veins at a dose of 0.2 mL/kg body weight [24–26].

### Image pre-processing

Patients from the Duke-Breast-Cancer-MRI dataset with NHG1 (*n* = 162) and NHG3 (*n* = 269) tumors were randomly divided into training (80%) and testing (20%) sets. External validation was performed using DCE MRIs of patients from the Advanced-MRI-Breast-Lesions dataset with NHG1 (*n* = 12) and NHG3 (*n* = 25) tumors. For each individual patient, min–max normalization was performed across the pre-contrast and the first three post-contrast phases. We cropped an area around each patient’s tumor under the guidance of a board-certified radiologist that was 3 × 14 × 64 × 64 (phase × superior/inferior × anterior/posterior × left/right). From each training patient’s set of 14 axial slices, four were randomly chosen for horizontal flipping, four for vertical flipping, with the remaining five unchanged. Each 3 × 64 × 64 (phases, anterior/posterior × left/right) axial slice was then used as an independent training input. In the testing set, the axial slice immediately superior to the center of the tumor was used without augmentation. Models were trained with 14, 12, 10, 8, 6, and 4 input slices per patient.

### Convolutional neural network (CNN) architecture

**Fig. 1:**
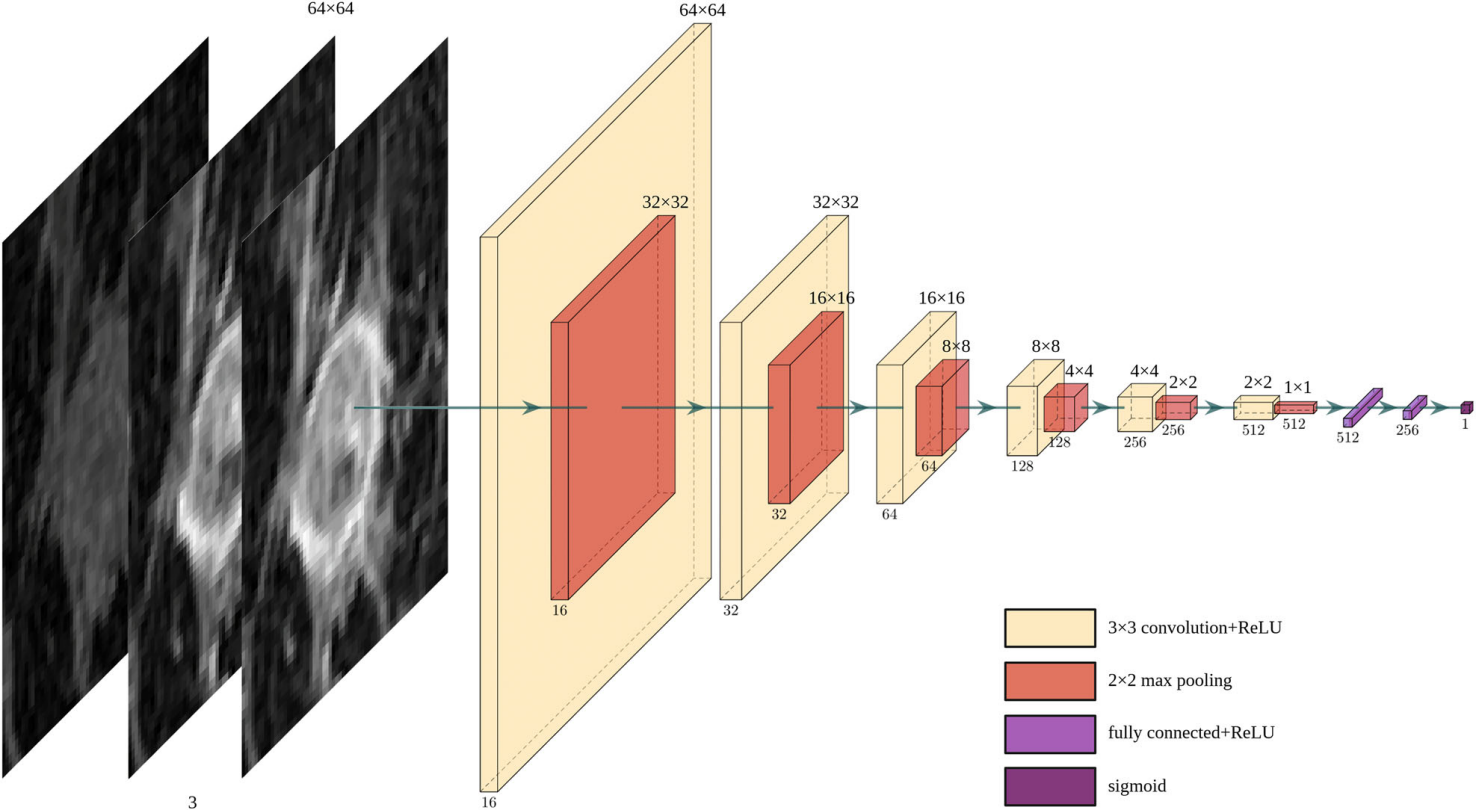
The architecture of DeepRadGrade (DRG), which was trained to perform binary classification of NHG 1 and 3 tumors. The 64 × 64 axial slices of three dynamic phases were used as input. The 3 × 3 convolution and padding of one were used to preserve, and 2 × 2 max pooling was used to downsample feature map dimensions. The number of feature maps was doubled at every layer, except in the first layer, where 16 feature maps were generated from the three input phases. A rectified linear unit activation function was used after every convolutional and fully connected layer. A sigmoid function was used on the output of the final node

Experimentation with more complex architectures, such as ResNet, 3-dimensional CNNs, and inputting the entire MRI, led to overfitting. Thus, a 2-dimensional approach with cropping was utilized. A sequential CNN was generated using two-dimensional 3 × 3 convolutional kernels (Fig. 1). After each convolutional layer, a rectified linear unit (ReLU) activation function and 2 × 2 max pooling were used to downsample image dimensions, with a doubling of the number of channels at every convolutional layer, yielding 512 features from a 1 × 1 matrix after the last convolutional layer. After flattening this tensor, two fully connected linear layers were used, with the first linear layer reducing features down to 256, followed by a ReLU activation function, and a final linear layer reducing features to a single node for binary classification. A sigmoid activation function was applied to the final output.

### Model training, testing, and validation

The CNN was trained to classify tumors as NHG1 or NHG3 using class-sensitive binary cross-entropy loss and the Adam optimizer for 1100 epochs at a batch size of 512 and a learning rate of 5 × 10^−6^. The model’s performance in distinguishing between NHG1 and NHG3 tumors was evaluated using the area under the receiver operator characteristic (ROC) curve (AUC) and its 95% confidence interval (CI). By maximizing the Youden index (sensitivity + specificity – 1), the optimal threshold for classification of NGH1-like or NGH3-like tumors was determined. The model then evaluated the 456 NHG2 tumors reserved as the test set from the Duke-Breast-Cancer-MRI dataset, which were then stratified according to the optimal threshold. NHG1-like tumors were called DeepRadGrade 2 negative (DRG2−), and NHG3-like tumors were called DRG2+, such that “higher-risk NHG2 tumors” were classified as DeepRadGrade 2 positive (NHG3-like) (DRG2+) and “lower-risk NHG2 tumors” as DRG2−. To better understand which image regions contributed to the CNN’s predictions, we applied gradient-weighted class activation mapping (Grad-CAM). Grad-CAM computes the gradients of the output class score with respect to the feature maps of a selected convolutional layer, producing a heatmap that highlights areas most influential for the model’s decision [27, 28]. We used the final convolutional block of our CNN (layer index 9) to generate Grad-CAM overlays, which were then rescaled and superimposed on the original pre- and post-contrast DCE-MRI slices.

### RFS analysis

Follow-up time was calculated in months from the breast cancer diagnosis date to the date of the first incident event (all-cause mortality or recurrence) or to the date of the last recurrence-free assessment (right censoring). Kaplan–Meier RFS curves with log-rank test were generated, and multivariate Cox proportional hazards modeling was used to compare the hazard ratio (HR) for risk of recurrence or all-cause mortality. Covariates adjusted for included the following variables at diagnosis: age, T stage ≥ 2, lymph node invasion (node [N] stage ≥ 1), and molecular subtype (categorized as ER+/HER2− (±PR), ER+/HER2+ (±PR), ER−/HER2+ (±PR), ER−/HER2− (PR−)). Among those with NHG2 tumors, two Cox proportional hazards models were generated, with and without the DRG classification as DRG2− or DRG2+.

### Statistical analysis

The predictive performance of Cox proportional hazards models was compared by applying a paired bootstrap (1000 resamples) to estimate each model’s concordance index (*C*-index). Mean difference and 95% CI of the bootstrap distribution were calculated, and a two-sided *p*-value was obtained. Differences in group characteristics were assessed using the chi-square test for categorical variables and the independent *t*-test for continuous variables, with *p*-values less than 0.05 being considered statistically significant.

### Software and hardware

Code for this study was written in Python 3.7.6 using the PyTorch, NumPy, Pandas, and LifeLines libraries and can be found at https://github.com/hadidchi/Deep-Rad-Grade. Training was performed on NVIDIA A1000 GPUs made available through the high-performance computing node cluster of the Albert Einstein College of Medicine.

## Results

**Fig. 2:**
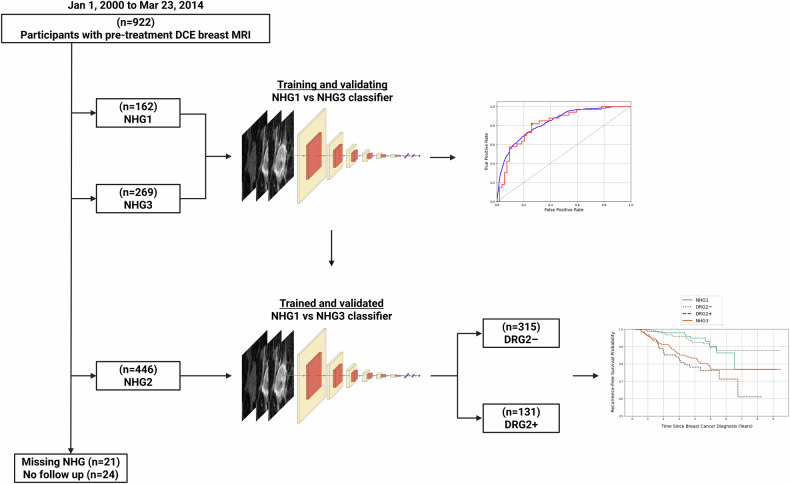
Patient selection flowchart and schematic overview of the training, validation, and application of the DRG model. DCE, dynamic contrast-enhanced; MRI, magnetic resonance imaging; NHG, Nottingham histologic grade

Figure 2 shows the patient selection flowchart. A CNN was trained to distinguish between DCE MRIs of 162 NHG1 and 269 NHG3 tumors and subsequently used to subdivide NHG2 tumors into low-and high-risk subgroups.

Several models were trained, using varying numbers of training slices around the tumor centroid per subject (Table 1). The model with eight axial slices per subject exhibited optimal performance (training AUC = 0.84 [95% CI: 0.83, 0.86], testing AUC = 0.82 [0.71, 0.91], external dataset validation AUC = 0.84 [0.69, 0.96]).

**Table 1:** Training, testing, and external dataset validation area under the receiver operating characteristic curve (AUC) in differentiating between grade 1 and 3 tumors with a varying number of axial slices included per subject

Slices per patient	Training AUC (*n* = 539)	Testing AUC (*n* = 108)	External dataset validation AUC (*n* = 37)
4	0.811 [0.789–0.834]	0.775 [0.658–0.869]	0.770 [0.585, 0.925]
6	0.785 [0.765–0.804]	0.745 [0.637–0.842]	0.782 [0.608, 0.921]
8	0.843 [0.828–0.857]	0.819 [0.714–0.911]	0.837 [0.686, 0.958]
10	0.839 [0.825–0.852]	0.811 [0.714–0.903]	0.821 [0.665, 0.950]
12	0.847 [0.835–0.858]	0.746 [0.633–0.850]	0.837 [0.667, 0.955]
14	0.859 [0.849–0.870]	0.729 [0.623–0.829]	0.798 [0.625, 0.931]

The Duke-Breast-Cancer-MRI dataset was used for training and testing, and the advanced-MRI-breast-lesions dataset was used for external validation

Using the optimal Youden’s threshold of 0.48, the trained network evaluated DCE MRIs of 456 patients with NHG2 tumors, classifying 315 as NHG1-like (DRG2−) and 131 as NHG3-like (DRG2+). Characteristics of NHG1, DRG2−, DRG2+, and NHG3 groups are shown in Table 2. At diagnosis, patients in the DRG2+ and NHG3 groups were ~50-years-old, and those in the NHG1 and DRG2− groups were ~55-years-old (*p* < 0.001). Compared to DRG2− patients, a higher proportion of DRG2+ patients were Black (27.48% vs 16.83%). T stage and N stage were generally higher in the DRG2+ group, while molecular subtype distribution and scores of individual NHG subcomponents, such as tubular formation, nuclear pleomorphism, and mitotic count, were largely similar among the DRG2− and DRG2+ groups.

**Table 2:** Baseline characteristics at breast cancer diagnosis of NHG and DRG groups

	NHG1 (*n* = 162)	DRG2− (*n* = 315)	DRG2+ (*n* = 131)	NHG3 (*n* = 269)
Age, mean ± SD	55.23 ± 9.87	55.67 ± 10.92	50.40 ± 10.88^†††^	49.96 ± 11.58
Race				
White	136 (83.95%)	242 (76.83%)	81 (61.83%)^††^	166 (61.71%)
Black	18 (11.11%)	53 (16.83%)	36 (27.48%)^†^	87 (32.34%)
Other race	8 (4.94%)	20 (6.35%)	14 (10.69%)	16 (5.95%)
Receptor status				
ER	155 (95.68%)	271 (86.03%)^**^	101 (77.10%)^†^	131 (48.70%)^‡‡‡^
PR	142 (87.65%)	246 (78.10%)^*^	90 (68.70%)^†^	97 (36.06%)^‡‡‡^
HER2	9 (5.56%)	53 (16.83%)^***^	25 (19.08%)	63 (23.42%)
Molecular subtype				
ER+/HER2− (±PR)	149 (91.98%)	234 (74.29%)^***^	88 (67.18%)	93 (34.57%)^‡‡‡^
ER+/HER2+ (±PR)	6 (3.70%)	37 (11.75%)^**^	13 (9.92%)	38 (14.13%)^‡‡‡^
ER−/HER2+ (±PR)	3 (1.85%)	16 (5.08%)	12 (9.16%)	25 (9.29%)
ER−/HER2− (PR−)	4 (2.47%)	28 (8.89%)^*^	18 (13.74%)	113 (42.01%)^‡‡‡^
T stage				
T 1	98 (60.49%)	173 (54.92%)	26 (19.85%)^†††^	91 (33.83%)^‡‡^
T 2	52 (32.10%)	121 (38.41%)	71 (54.20%)^††^	139 (51.67%)
T 3	11 (6.79%)	17 (5.40%)	28 (21.37%)^†††^	27 (10.04%)^‡‡^
T 4	1 (0.62%)	1 (0.32%)	6 (4.58%)^††^	12 (4.46%)
T X	0 (0.00%)	3 (0.95%)	0 (0.00%)	0 (0.00%)
N stage				
N 0	117 (72.22%)	193 (61.27%)^*^	62 (47.33%)^††^	143 (53.16%)
N 1	34 (20.99%)	87 (27.62%)	46 (35.11%)	83 (30.86%)
N 2	4 (2.47%)	13 (4.13%)	11 (8.40%)	28 (10.41%)
N 3	5 (3.09%)	14 (4.44%)	8 (6.11%)	10 (3.72%)
N X	2 (1.23%)	8 (2.54%)	4 (3.05%)	5 (1.86%)
M stage				
M 0	129 (79.63%)	240 (76.19%)	94 (71.76%)	200 (74.35%)
M 1	1 (0.62%)	5 (1.59%)	7 (5.34%)	5 (1.86%)
M X	32 (19.75%)	70 (22.22%)	30 (22.90%)	64 (23.79%)
Tubular formation				
1	63 (38.89%)	3 (0.95%)^***^	2 (1.53%)	0 (0.00%)
2	72 (44.44%)	52 (16.51%)^***^	15 (11.45%)	4 (1.49%)^‡‡‡^
3	27 (16.67%)	260 (82.54%)^***^	114 (87.02%)	265 (98.51%)^‡‡‡^
Nuclear pleomorphism				
1	52 (32.10%)	0 (0.00%)^***^	1 (0.76%)	0 (0.00%)
2	109 (67.28%)	205 (65.08%)	70 (53.44%)^†^	9 (3.35%)^‡‡‡^
3	1 (0.62%)	110 (34.92%)^***^	60 (45.80%)^†^	260 (96.65%)^‡‡‡^
Mitotic count				
1	158 (97.53%)	266 (84.44%)^***^	113 (86.26%)	0 (0.00%)^‡‡‡^
2	4 (2.47%)	46 (14.60%)^***^	17 (12.98%)	126 (46.84%)^‡‡‡^
3	0 (0.00%)	3 (0.95%)	1 (0.76%)	143 (53.16%)^‡‡‡^

*SD* standard deviation, *ER* estrogen receptor, *PR* progesterone receptor, *HER2* human epidermal growth factor receptor 2, *X* stage unknown, *cm* centimeters, *IQR* interquartile range

^*^ *p* < 0.05, ^**^ *p* < 0.01, and ^***^ *p* < 0.001 between NHG1 and DRG2−

^†^ *p* < 0.05, ^††^ *p* < 0.01, and ^†††^ *p* < 0.001 between DRG2− and DRG2+

^‡^ *p* < 0.05, ^‡‡^ *p* < 0.01, and ^‡‡‡^ *p* < 0.001 between DRG2+ and NHG3

**Fig. 3:**
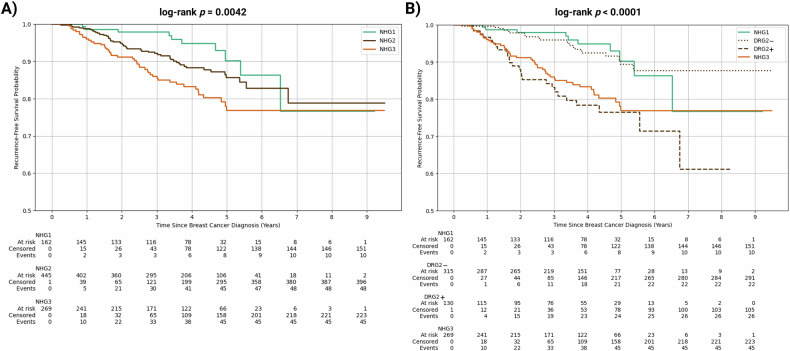
Kaplan–Meier RFS curves for NHG groups (**A**) before and (**B**) after stratification of the NHG2 group by DRG

Figure 3A shows the Kaplan–Meier RFS curves, in which the NHG2 group had an intermediate prognosis between that of the NHG1 and NHG3 groups. After applying DL risk stratification, DRG2− group showed similar RFS as the NHG1 group, and the DRG2+ group showed similar RFS as the NHG3 group (Fig. 3B).

Table 3 shows multivariate Cox-proportional hazards models evaluating RFS among patients with NHG2 breast cancer. The model using traditional prognostic factors (age, lymph node invasion, T stage, and molecular subtype) yielded a *C*-index of 0.68 [0.59 0.76], with the dominant predictors being lymph node invasion (adjusted HR = 1.64 [0.92, 2.93]), T stage ≥ 2 (adjusted HR = 3.49 [1.72, 7.08]), and triple negative vs ER+/HER2− (±PR) molecular subtype (adjusted HR = 2.48 [1.21, 5.06]). After adding the DRG classification of DRG2+ or DRG2− to the model, *C*-index improved from 0.68 [0.61, 0.76] to 0.73 [0.66, 0.80] (*p* = 0.040). Being classified as DRG2+ by the DL model was a significant predictor of RFS (adjusted HR = 2.39 [1.29, 4.45]), independent of age at diagnosis, lymph node status, T stage, and molecular subtype.

**Table 3:** Multivariate Cox-proportional hazards (CoxPH) models and their concordance indices (*C*-index) modeling risk of RFS (A) without and (B) with DRG classification

	(A) Without stratification		(B) With stratification	
CoxPH *C*-index [95% CI]	0.68 [0.61, 0.76]		0.73 [0.66, 0.80]	
*C*-index *p*-value	0.040			
Covariate	Adjusted HR [95% CI]	*p*-value	Adjusted HR [95% CI]	*p*-value
DRG2+ vs DRG2−	−	−	2.39 [1.29, 4.45]	0.0059
Age at diagnosis (years)	1.01 [0.99, 1.04]	0.32	1.02 [0.99, 1.05]	0.12
Lymph node invasion	1.64 [0.92, 2.93]	0.096	1.53 [0.85, 2.75]	0.15
T stage ≥ 2	3.49 [1.72, 7.08]	< 0.005	2.64 [1.25, 5.55]	0.011
ER+/HER2+ (±PR) vs ER+/HER2− (±PR)	0.71 [0.25, 2.04]	0.53	0.74 [0.26, 2.11]	0.57
ER−/HER2+ (±PR) vs ER+/HER2− (±PR)	0.67 [0.16, 2.85]	0.59	0.67 [0.16, 2.83]	0.58
ER−/HER2− (PR−) vs ER+/HER2− (±PR)	2.48 [1.21, 5.06]	0.013	2.17 [1.06, 4.47]	0.035

*CI* confidence interval, *HR* hazard ratio

Grad-CAM showed that the CNN attended to the enhancing tumor region and adjacent peritumoral tissue (Supplementary Fig. 1). Shown are two representative cases of NHG2 tumors, one classified as DRG2+ and DRG2−.

## Discussion

We developed and validated a DL framework using DCE MRI to refine risk stratification among NHG2 tumors. Incorporating this classification into the predictive Cox-proportional hazards model of RFS improved the *C*-index from 0.68 to 0.73 (*p* = 0.040). Patients with DRG2+ classification were more likely than DRG2− patients to experience recurrence or mortality (adjusted HR = 2.39 [1.29, 4.45]). This NHG2 sub-stratification provided added prognostic value, independent of age, lymph node invasion, T stage, and molecular subtype.

Our study was inspired by prior efforts utilizing genomic profiling and histopathological images to refine tumor grading. Wang et al used a 34-gene RNA sequencing panel to up- and down-grade transcriptomic grade tumors, finding that the high transcriptomic grade subgroup within NHG2 tumors had a significantly higher recurrence or all-cause mortality risk than the low transcriptomic grade subgroup (HR = 2.43 [1.13, 5.20]) [8]. Another study applied a 97-gene signature to stratify NHG2 tumors into low- and high-risk subgroups, reporting that a high gene expression grade index was associated with an increased recurrence risk (HR = 3.61 [2.25, 5.78]) [7]. EndoPredict, an RNA sequencing-based approach, dichotomized ER + HER2 − NHG2 breast cancer patients and found a substantial difference in RFS (HR = 5.07 [3.23, 7.94]) [10]. In histopathology, a DL analysis of whole-slide images showed an increased risk of recurrence or all-cause mortality in the up-graded NHG2 group compared to the down-graded NHG2 group (HR = 1.91 [1.11, 3.29], *p* = 0.019) [14]. Our study is the first to apply DL-based analysis of DCE MRI for up- and down-grading of NHG2 tumors. We leveraged multiple DCE time points, capturing temporal contrast dynamics that provide important information about tumor pathophysiology and vascularity [29]. Because DCE MRI is already part of standard diagnostic workflows, this approach can be integrated into clinical practice at no additional cost. We also incorporated this DL-based stratification into a multivariate predictive model of RFS, to show that NHG2 sub-stratification provided added prognostic value, independent of age, lymph node invasion, tumor T stage, and molecular subtype. This framework can complement existing genomic and histopathological methods to further refine prognosis among this heterogeneous NHG2 group.

Our framework has the potential to become a tool that synergistically augments existing genomic and pathological methods. Genomic assays such as Oncotype Dx and Ki-67 proliferation index provide molecular insights into tumor biology [30–32], whereas our MRI-based DL approach leverages routinely acquired imaging to offer additional, non-invasive prognostic information. Importantly, these modalities capture different dimensions of tumor heterogeneity and may be more powerful when considered together [33, 34]. By integrating imaging-based risk stratification with molecular assays together, researchers and clinicians could achieve a more holistic assessment of tumor aggressiveness while also balancing considerations of cost, accessibility, and patient convenience.

Improved sub-stratification has significant clinical implications. Patients in the high-risk DRG2+ subgroup may benefit from more aggressive treatment or closer surveillance to mitigate their increased recurrence risk, while those in the low-risk DRG2− subgroup could potentially avoid overtreatment and its associated toxicities [22, 23]. This refined classification supports the growing emphasis on personalized cancer therapy, where enhanced prognostic precision informs treatment decisions regarding chemotherapy, targeted therapy, and radiation [35].

Several limitations should be acknowledged. This was a retrospective analysis, and variations in MRI acquisition parameters, manufacturer, and field strength across institutions and scanners, which were not directly adjusted for, may impact the performance of our DL model. The external data set included only 37 patients in whom follow-up mortality or recurrence data were not available. Additional testing on larger and more granular datasets is needed to improve generalization. Another important limitation is that our CNN framework was not benchmarked against radiologists or widely used clinical risk models such as Oncotype Dx, Ki-67 proliferation index, or genomic assays. Direct comparison to these methods was not feasible because the majority of patients in the Duke dataset lacked genomic information, and radiologist grading data were not systematically available. As such, while our model demonstrates promising prognostic discrimination, its interpretability and clinical integration remain uncertain without head-to-head evaluation against current standards of care. Future work should incorporate prospective comparisons with human readers and genomic assays to better establish the complementary role of DL within existing clinical workflows. The model was trained using NHG1 and NHG3 labels derived from summing three histological scores. However, NHG grading is subject to high inter-observer variability without consensus review. Thus, there is the risk of noisy or inconsistent labels in the training process. As a proof-of-concept study, eight slices around the center of the tumor were found to be the optimal input for the model (Table 1). This is likely because slices outside of this volume like to be noisy and do not contain “useful” information. Moreover, using 8 slices also reduced computational cost. The proportion of patients who were Black in the DRG2+ group was higher than that in the DRG2− group (27.48% vs 16.83%). Race could be a confounding predictor due to its association with triple-negative breast cancer. In our multivariate analysis, relevant confounding variables other than race were adjusted for. Similarly, our analysis did not adjust for treatment type (neoadjuvant chemotherapy vs primary surgery) as this variable is heavily correlated with already adjusted-for covariates such as molecular subtype. Our model used only DCE MRI. Incorporating additional MRI protocols, such as diffusion-weighted and T2-weighted imaging, or integrating histopathological data with prospective multicenter validation could enhance performance and utility. While we employed a conventional CNN that could be readily implemented, more advanced DL architectures and fine-tuning of more complex models on larger datasets can be explored [36].

## Conclusion

We developed and validated a DL DCE MRI-based classification framework to stratify grade NHG2 breast tumors. The integration of DL-driven DCE MRI analysis into clinical workflows has offered oncologists a powerful, cost-effective tool to tailor treatment strategies more effectively, which has the potential to improve clinical outcomes through individualized risk stratification. As imaging technologies and artificial intelligence continue to evolve, the use of DL models in cancer prognostication represents a promising frontier in precision oncology.

## Supplementary information

Supplementary information

## Abbreviations

AUC: Area under the receiver operating characteristic curve
CI: Confidence interval
*C*-index: Concordance index
CNN: Convolutional neural network
DCE: Dynamic contrast-enhanced
DL: Deep learning
DRG: DeepRadGrade
DRG2−: DeepRadGrade 2 negative (NHG1-like)
DRG2+: DeepRadGrade 2 positive (NHG3-like)
ER: Estrogen receptor
HER2: Human epidermal growth factor receptor 2
HR: Hazard ratio
MRI: Magnetic resonance imaging
NHG: Nottingham histologic grade
PR: Progesterone receptor
RFS: Recurrence-free survival
