# Supplementary material for: A deep learning framework to stratify Nottingham histologic grade 2 breast tumors based on dynamic contrast-enhanced MRI
Source: Eur Radiol. 2025 Dec 17;36(5):3335–45. doi: 10.1007/s00330-025-12208-6 (PMC13086883; doi:10.1007/s00330-025-12208-6)

A Deep Learning Framework to Stratify Nottingham Histologic Grade 2 Breast Tumors Based on Dynamic Contrast Enhanced MRI

ELECTRONIC SUPPLEMENTARY MATERIAL

Eur Radiol (2025) Hadidchi R, Agrawal A, Liu MZ, et al.

Supplementary Figure 1. Representative Gradient-weighted class activation mapping visualization for two patients with Nottingham Histological Grade 2 tumors reclassified as **A)** Deep Rad Grade 2+ (higher-risk) and **B)** Deep Rad Grade 2− (lower-risk) by the model.

A)

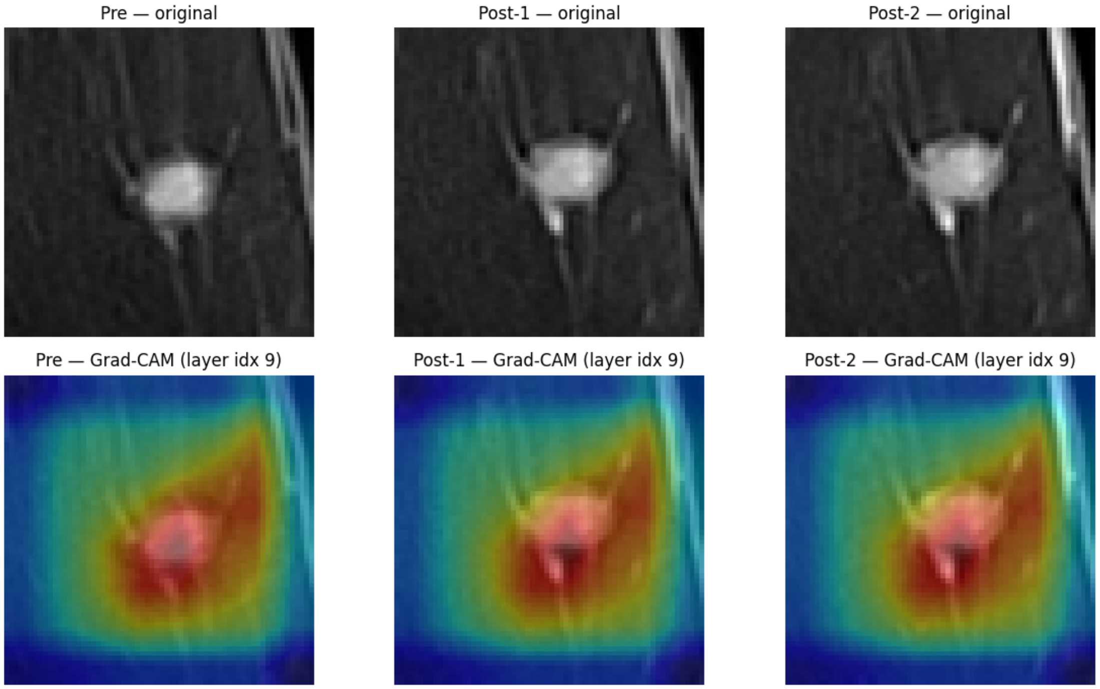

B)

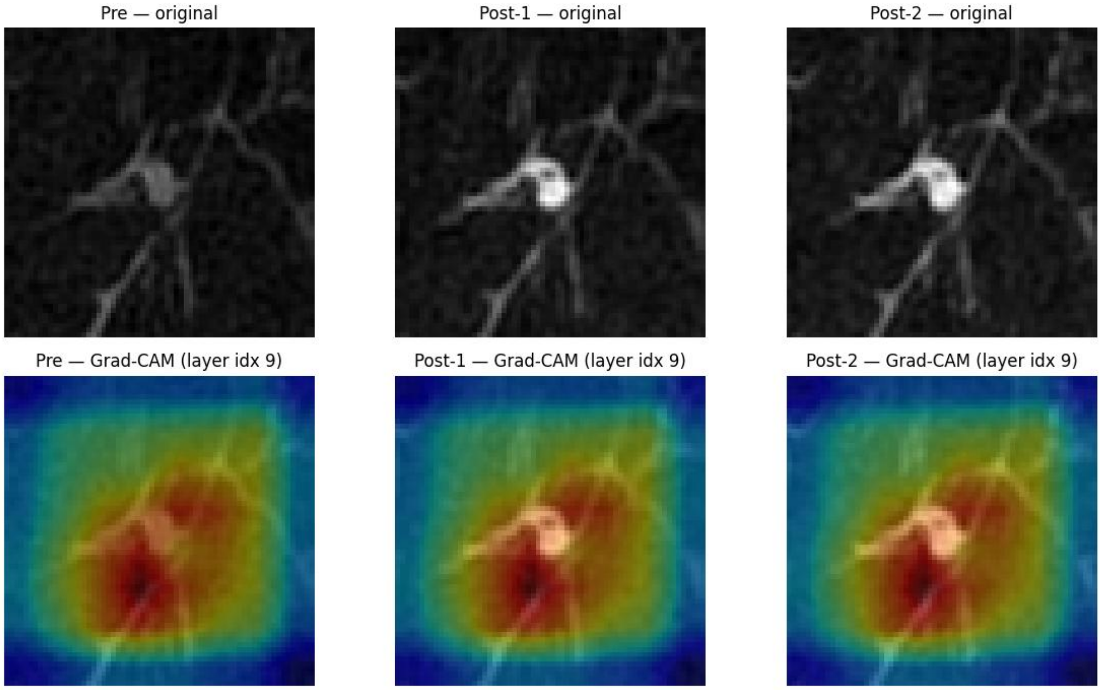

Supplement: Supplementary file 1 — Supplementary information [file 330_2025_12208_MOESM1_ESM.pdf]
